# Recovery of resources from industrial wastewater employing electrochemical technologies: status, advancements and perspectives

**DOI:** 10.1080/21655979.2021.1946631

**Published:** 2021-08-01

**Authors:** Viralkunvar Devda, Kashika Chaudhary, Sunita Varjani, Bhawana Pathak, Anil Kumar Patel, Reeta Rani Singhania, Mohammad J. Taherzadeh, Huu Hao Ngo, Jonathan W. C. Wong, Wenshan Guo, Preeti Chaturvedi

**Affiliations:** a Paryavaran Bhavan, Gujarat Pollution Control Board, Gandhinagar, Gujarat, India; b School of Environment and Sustainable Development, Central University of Gujarat, Gandhinagar, Gujarat, India; c Department of Marine Environmental Engineering, National Kaohsiung University of Science and Technology, Kaohsiung City, Taiwan; d Swedish Centre for Resource Recovery, University of Borås, Borås, Sweden; e Centre for Technology in Water and Wastewater, School of Civil and Environmental Engineering, University of Technology Sydney, Sydney, NSW, Australia; f Institute of Bioresource and Agriculture and Department of Biology, Hong Kong Baptist University, Kowloon Tong, Hong Kong, HKSAR; g Environmental Toxicology Group, Aquatic Toxicology Laboratory, Council of Scientific and Industrial Research-Indian Institute of Toxicology Research (CSIR-IITR), Lucknow, Uttar Pradesh, India

**Keywords:** Industrial wastewater, effluent, health hazards, biological treatment, electrochemical technology, resources

## Abstract

In the last two decades, water use has increased at twice the rate of population growth. The freshwater resources are getting polluted by contaminants like heavy metals, pesticides, hydrocarbons, organic waste, pathogens, fertilizers, and emerging pollutants. Globally more than 80% of the wastewater is released into the environment without proper treatment. Rapid industrialization has a dramatic effect on developing countries leading to significant losses to economic and health well-being in terms of toxicological impacts on humans and the environment through air, water, and soil pollution. This article provides an overview of physical, chemical, and biological processes to remove wastewater contaminants. A physical and/or chemical technique alone appears ineffective for recovering useful resources from wastewater containing complex components. There is a requirement for more processes or processes combined with membrane and biological processes to enhance operational efficiency and quality. More processes or those that are combined with biological and membrane-based processes are required to enhance operational efficiencies and quality. This paper intends to provide an exhaustive review of electrochemical technologies including microbial electrochemical technologies. It provides comprehensive information for the recovery of metals, nutrients, sulfur, hydrogen, and heat from industrial effluents. This article aims to give detailed information into the advancements in electrochemical processes to energy use, improve restoration performance, and achieve commercialization. It also covers bottlenecks and perspectives of this research area.

## Graphical abstract

**Figure:**
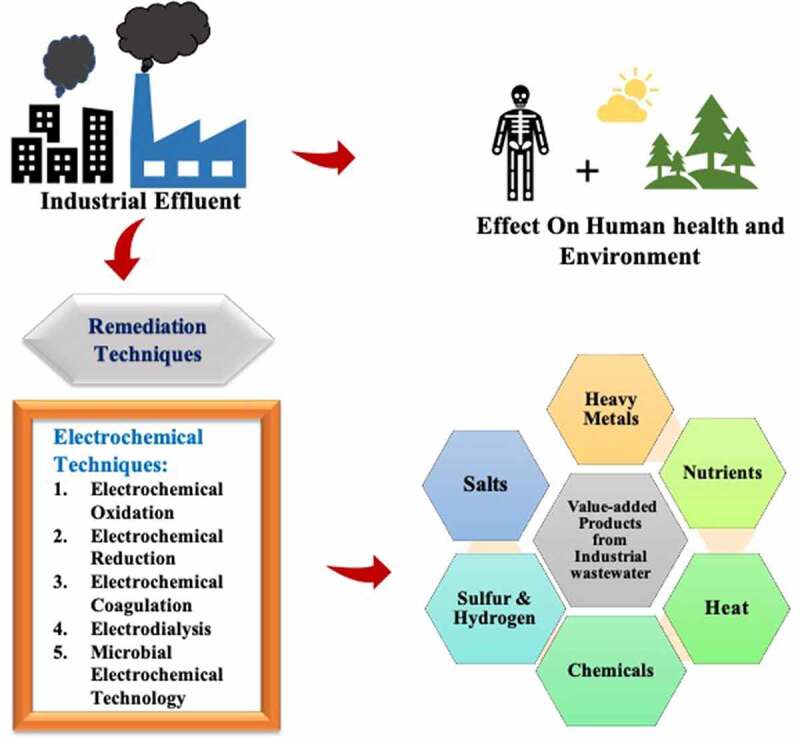

## 1. Introduction

**Figure 1.**
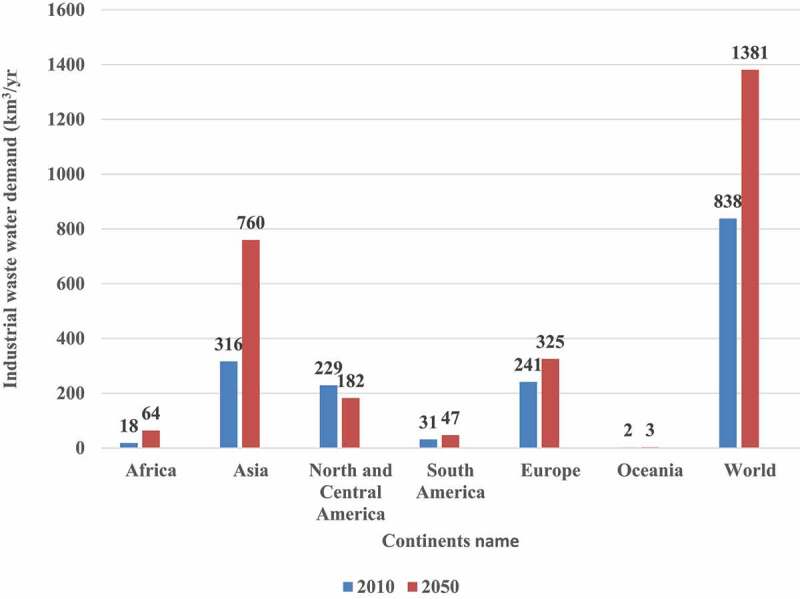
Industrial water demand, by continent, 2010 and 2050

Water use has increased at twice the rate of population growth over the previous century (FAO,2013). The urban water supply is vulnerable because of increasing urbanization and the high population density of cities. Climate change is estimated to result in an additional 10% decrease in freshwater supply for 685 million people residing in over 570 cities by 2050. Figure 1 shows Industrial wastewater demand by continents from 2010–2050 [1].

**Figure 2.**
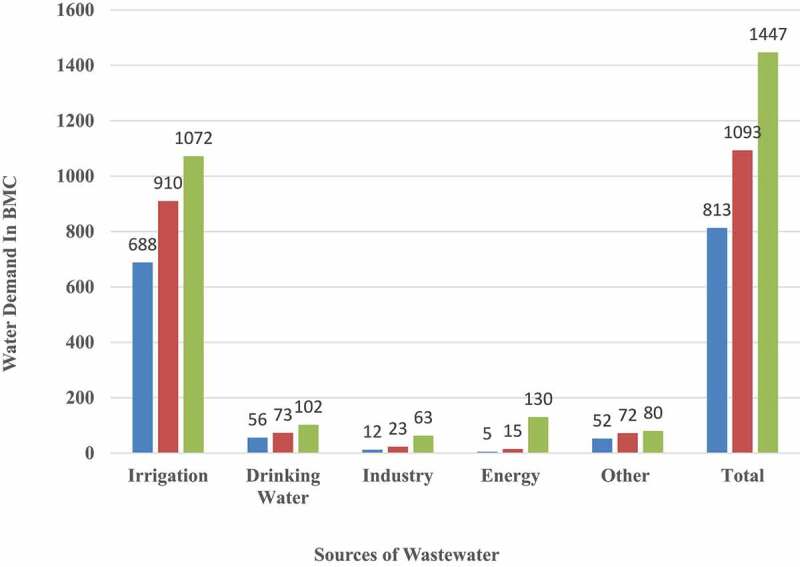
Projected Water Demand in India (Water Demand in Billion/m^3^)

Wastewater is composed of 1% suspended and dissolved solids and 99% water [2]. The concentration of pollutants such as heavy metals, diseases, pesticides and fertilizers, organic waste, and new contaminants has been increasing in the world’s freshwater resources [3]. Organic matter in water pollution is increasing due to increased industrial and municipal wastewater discharge, decreased runoff, and reduced water dilution capacity, and agricultural intensification [4]. In 2012, the organization for economic co-operation and development (OECD) projected that between 2000 and 2050 water demand would be increased by 55% globally [5]. The use of water and wastewater is responsible for 3–7% of GHG emissions [6,7]. Globally, more than 80% of the wastewater is neither collected nor treated and is released into the environment without proper treatment, with only 8% of industrial and municipal wastewater is treated [7]. High-income countries treat around 70% of wastewater they generate, in middle-income countries this ratio falls to 38%, whereas in lower-middle-income countries, it rises to 28%. The industrialization process is adversely affecting the global environment [2]. The release of improperly treated wastewater into the environment causes several health impacts on human health such as the enhanced burden of diseases because of decreased drinking and bathing water property and direct impact on the environment such as decreased biodiversity, bioaccumulation of toxins, increased GHG emissions, degrade aquatic ecosystem and increased water temperature and economic productivity such as reduced industrial and agricultural production, the lower market price of harvest crops, etc. Wastewater is mainly originating from domestic and industrial sectors while other sources are urban runoff, agricultural runoff, mining activities, landfill leachate, municipal, and energy generation [8]. These wastewater sources include hazardous organic components like persistent organic pollutants, hydrocarbons, chlorinated solvents, PCBs, and volatile organic compounds [9]. The industrialization process is adversely affecting the global environment [2]. Some small-scale industries are not permitted to build or operate wastewater treatment plants, limiting their restriction to regulate pollution. Common effluent treatment plants (CETPs) are regarded as one of the feasible wastewater treatment solutions for small and medium-sized businesses. CETPs are treatment systems of collective effluents from industries and get potential benefits in terms of environmental improvements and pollution reduction. There are 192 CETPs established in different states of India. [CETP 10]. There are mainly two types of wastewater treatment and collection system: (a) Offsite system, where wastewater is transported into a treatment plant through a sewerage network, and (b) On-site system, where wastewater is amassed in a septic tank and this tank can be opened in another location. Figure 2 shows the projected water demand in India up to 2050.

Electrochemical technology is used for the treatment of wastewater. Without the addition of chemicals; nutrients, hydrogen, sulfur, metallic ions, and chemical components can be retrieved through EC precipitation, EC oxidation-reduction, electrochemical stripping, and electrochemical membrane processes [11,12]. Both membrane and biological processes enhance the efficiency and cleanliness of products [13–15]. The increasing population has led to serious pollution of the environment such as deficiency of water and resource storage worldwide [16]. Now a day’s nitrogen and phosphorus are critical agricultural fertilizers [17]. Artificial production of nitrogenous fertilizers is energy-consuming and phosphorus generation will run out in the next decade [18–20]. As a result, recovering nitrogen and phosphorus from wastewater is gaining popularity. Attractive advantages of electrochemical technologies such as ease of maintenance, no addition of chemicals, high efficiency, flexibility, little sludge, by-products, and the possibility for energy and resource recovery have been used in the remediation of wastewater [12,21,22]. There are some electrochemical technologies used for the treatment of wastewater such as refractory organics degradation by electrochemical oxidation, precious metal deposition, denitrification by desalination and electrocoagulation, and reusing water by electrodialysis [23–25].

An MFC is a system that generates electricity from biomass using bacteria [26]. Nutrients like phosphorus can be recovered from sludge by using the supercritical water oxidation technique. Metal Sulfides can be recovered by using sulfate-reducing bacteria [27] and metals like Cr and Cu can be recovered by using electrodialysis [28]. Salts like sodium sulfate, sodium carbonate, and potassium nitrate can be recovered by using osmotic membrane contactors treatment [17]. Microalgae such as Chlorophyceae and *Chlorella vulgaris* are used for biofuel production and also used for the production of carbohydrates, proteins, and vitamins [29, 30; 31]. Using these recovered resources in a different field reduces the use of hazardous substances or chemicals in industry, and lowers the cost of energy production. Resource recovery from wastewater consists of only a few amounts of pollutants in water that can be less harmful to the environment and human health. After resource recovery from wastewater, only a small portion of this wastewaters is used in a planned and safe way.

The present review intends to expand the literature about developments in recovering resources from wastewater through electrochemical techniques. It gives a brief idea about the need for recovery of resources and hazards associated with the pollutants present in the wastewater. It also covers knowledge gaps and future perspectives of this research area.

## 2. Hazards of pollutants from industrial wastewater

Rapid industrialization has a dramatic effect on developing countries leading to significant losses to economic well-being in terms of toxicological impacts on humans and the environment via air and soil pollution [32]. Human-induced pollution affects the world’s natural water resources to the extent that it becomes impossible to reestablish pristine conditions [33–35]. When industrial effluent is discharged into bodies of water without being properly treated, it causes serious water contamination. Because of high biochemical oxygen demand, chemical oxygen demand values, high levels of sulfate, nitrogen, and phosphate, it induces eutrophication of polluted water resources [36, 37; 38]. Industrial wastewater is also responsible for acidification and soil pollution in the case of hydrocarbon emission [31,39]. Furthermore, various researchers have reported that it inhibits seed germination, causes Mn deficiency in the soil, increases soil acidity, and decreases the yield and growth of cultivated plants. Highly carcinogenic chlorine compounds identified in industrial effluents include highly carcinogenic dioxins, organic acids, and furan. They are mutagenic and bio-accumulative in plants and animals when exposed to their environmental condition [33, 37, 40]. Various treatment technologies are developed for the treatment of pollutants found in the water [41]. Table 1 shows the treatment technologies of water-borne contaminants.

**Table 1.** Treatment technologies for water-borne contaminants

Water-borne contaminants	Treatment technologies
Heavy metals	Chemical precipitation
Settleable solids	Sedimentation Screen filter Sand filtration
Iron and manganese	Chemical oxidation Biological filters
Arsenic	Ion exchange Chemical precipitation and Activated carbon adsorption Membrane filtration
Organic compounds	Chemical oxidation Activated carbon adsorption
Nitrogen compounds	Stripping (suited for ammonia only) Ion exchange Membrane filtration and Biological filters
Salinity	Thermal processes (e.g., solar still) Dilution with rainwater Ion exchange Membrane filtration
Colloids	Coagulation and flocculation
Fecal bacteria	disinfection
Cyanobacteria [or another type of algal bloom)	Chemical oxidation Coagulation flocculation Sand filtration Micro-screen filter

Pollution control activities in India are shared by three separate ministries: The Ministry of Environment, Forests, and Climate Change (MoEF & CC), the Ministry of Housing and Urban Affairs (MoHUA), and the newly created Ministry of Jal Shakti. The MoEF & CC is the nodal body, and it, along with the Central Pollution Control Board (CPCB), is in charge of establishing policies, laws, and related standards. Regulatory laws are used by institutions to carry out their duties. The Water Prevention and Control of Pollution Act was passed in 1974 as the first law for the prevention and control of water pollution, and it resulted in the creation of responsible bodies for enforcement at the federal and state levels. The National Water Policy (NWP) was published in 2012. It recommends water recycling and reuse, as well as return, flows for demand control and effective water usage, as well as rewards by efficient water pricing [42].

To avoid or reduce contamination from non-treated or partially treated industrial effluent, all WEPA (Water Environment Partnership in Asia) partner countries (Cambodia, China, Indonesia, Japan, Republic Korea, Loa PDR, Malaysia, Myanmar, Nepal, Philippines, Sri Lanka, Thailand, and Viet Nam) have legislation in place, and all countries, apart from Myanmar, have established wastewater requirements that the industrial zone should meet. The Environmental Impact Assessment (EIA) is a method for preventing emissions. Laws or regulations in Indonesia, Japan, and Vietnam have recently been revised. Impact Assessment in Cambodia is used to prevent the launch of operational capabilities. To fix them, all countries have already implemented inspection programs, governmental directives, and punishments [43]

In Europe, the discharge of industrial effluent is regulated both explicitly as a portion of the environmental law on industry and indirectly by European policies that address water problems on a broad scale. Specific directives govern aspects of industrial effluent generation and management under the Water Framework Directive (WFD, 2000/60/EC). The Urban Wastewater Directive (UWWTD, 91/271/EEC), the Groundwater Directive (2006/118/EC), and the Environmental Quality Standards Directive (2008/105/EC) have been the most important. The Industrial Emissions Directive (IED, 2010/75/EU) regulates the direct and indirect release of pollutants into the atmosphere by industry. In Europe, the Industrial Emission Directive currently controls 31 industrial sectors and over 50, 000 installations. All of these devices, when combined, form the key mechanism for industrial wastewater control, and every one governs a particular element of the different routes by which industrial wastewater may be emitted [M. 44].

## 3. Treatment technologies

Treatment of wastewater is a mixture of physical, chemical, and biological techniques used to eliminate contaminants from wastewater [38,45,46].

### 3.1. Physical

In the physical process, natural forces are applied to remove contaminants. There are mainly three types of physical methods [Y. 47; 48], (1) Flow equalization: It is used to enhance the efficiency of secondary wastewater processes by flattening out operation characteristics like pollutants levels, temperature, and flow over a period [R. 49]. (2) Sedimentation: It is often known as settling, is the removal of particulate matter, grit in the primary settling basin, and the flow of chemicals when the chemical coagulation method is used [50]. (3) Flotation: In the flotation process gas bubbles are introduced to get rid of liquid or solid particles from a liquid [51–53]. Flotation is also commonly used in industrial WWTPs to eliminate grease, oils, fats, and suspended particulates from wastewater. These are known as dissolved air flotation units. DAF machines, in specific, are used to remove oil from the effluents of oil refineries, chemical and petrochemical industries, natural gas treatment plants, and other similar industrial sites.

The main advantage of physical methods is they can be easily integrated with chemical methods. They are useful for primary clarity, metal discrimination, and short retention time. Also, there are some limitations associated with these technologies like high initial capital expenditure, costs of energy, the costs of maintenance [54,55]

### 3.2. Mechanical

There are mainly two types of mechanical methods, (1) Screening: The initial stage in any wastewater treatment system is screening. This procedure entails removing big floating and non-biodegradable materials that regularly penetrate a wastewater treatment plant, such as clout, papers, tins, woods, and plastics. This method removes overall contaminants from the waste stream to safeguard downstream devices from damage and prevent the suspended materials from entering the primary settling tank [56,57]. [2) Filters: Filters are used in biological methods to encouraged aerobic attached-growth to remove organic materials from effluent.

The major advantages of mechanical methods are water filtration is inexpensive, and that it doesn’t require a huge amount of money to keep it running; the water’s smell and flavor will increase; water filtering also removes chlorine from hard water, and in addition, the process ensures that dangerous pollutants are eliminated from the water. Also, some limitations are there like, the filtrate doesn’t remove all pathogens and pollutants, when the procedure is running, very minute particles can pass via the membranes used to filter water, the greater frequency of raking raises labor expenditures, and throughout cleaning, removing this mat may generate flow spikes, which can lower the solid-holding capacity of downstream units 54, 55].

### 3.3. Biological

Biological treatment is also known by the name of secondary treatment [58]. Biological phenomena like bioremediation are an eco-friendly technique for removing color from effluents with low cost and optimal working time [22; 59]. The combined activity of biological substances like fungi, algae, yeast, and bacteria can disintegrate and absorb the diversity of contaminants [58,60]. The biological techniques used to degrade effluents were successfully applied. The biological breakdown is economically viable, environmentally responsible, and results in decrease sludge quantity than other technologies [61,62].

#### 3.3.1. Aerobic treatment

These processes happen when oxygen is present and generates cell energy through the use of aerobic respiration. There are major three aerobic treatment technologies: (A) Activated sludge process: In this process, the dispersed growth reactor is an aeration tank with a slurry suspension and microorganisms. As a result of the sedimentation process, these microorganisms are isolated from the fluid and the purified liquid is a secondary effluent. To maintain a high standard of mixed-liquor suspended solids, a fraction of the biological sludge is retrieved to the aeration basin. To keep a roughly constant saturation of microbes in the unit, the residue is taken from the process and transferred to sludge treatment. (B) Trickling filters: A trickling filter is an attached growth technique in which microbes that are amenable for treatment are connected to an inert packing substance. These are occupied with substrates such as plastic forms, stones, or wood inclined. The buoyed matter is isolated from the fluid by a secondary clarifier and the slurry treatment is evacuated. The purpose of the trickling filter is to convert dissolved and unsettled organic material biologically and remove it through sedimentation. (C) Rotatory bio contactor: It is also known as a fixed film reactor. It is equal to bio-filters so far as microorganisms are fixed to sustain the environment. In the rotating biological contactors, the holder is a slowly rotating disk and oxygen is transferred into the wastewater through the rotation of the disk which creates surface turbulence [58,63–65].

High treatment efficiencies for COD, BOD, TSS, P, and N; operating environments are highly adaptable, excellent effluent consistency, it is possible to generate electricity from biogas and there is no need for specialized staff. These are the major advantages of the aerobic treatment process, and there are some limitations like pathogen elimination is minimal; reliance on a continuous power supply; high upkeep specifications; susceptible to toxic shock loads, and regular failure of critical parts such as shafts, bearings, drives, and discs if not built to a high standard [66,67]

#### 3.3.2. Anaerobic treatment

These processes are occurring in absence of oxygen and produce biogas as a by-product and also produce biosolids by processing. In anaerobic treatment, the up-flow anaerobic blanket reactor is used [L. 68]. It is a self-contained cell system made up of sludge cover in a lower layer and a higher liquid layer. It is used to break down the waste pulp and to gain biogas generation in a small size anaerobic reactor [64,69]. The treatment technique requires little energy and few nutrients. The major advantages of the anaerobic treatments are the minimal sludge output; low nutrient requirements; low initial and ongoing capital and operating costs; and methane production as an energy source. Also, there are some major limitations like an extensive startup and retention periods; needs high temperatures to function properly; needs for control to ensure proper operation; and shock and varying loads can disrupt microbial equilibrium [55,66,67].

#### 3.3.3. Anoxic treatment

**Figure 3.**
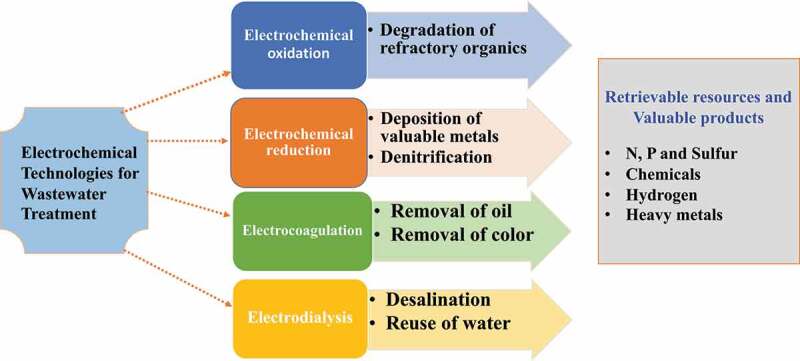
Electrochemical treatment technologies for industrial wastewater

These processes happen when oxygen is not available and generate energy via aerobic respiration. The primary goal of anoxic treatment is to eliminate N and P from effluents prior to they are discharged to the receiving water body. Therefore, to avoid eutrophication of rivers and creeks, anoxic treatment of effluent is required to overcome N and P contents in the wastewater until an allowable level earlier release to surface water is achieved. Nutrient removal improves the functioning of the processing station; it becomes more compact and loses the growth of filamentous organisms because of O_2_ deficiency; limited sludge is generated because the NO_3_^−^ created in the anoxic zone may be utilized to eliminate biochemical oxygen demand in the aerobic zone [64,70]. Figure 3 shows Electrochemical treatment technologies for industrial wastewater.

## 4. Electrochemical technologies for the treatment of wastewater

### 4.1. Electrochemical oxidation and reduction

Electrochemical-oxidation is a potent method to decompose and mineralize strong organic compounds [25,71,72]. Electrochemical oxidation processes are usually classified as directly and indirectly oxidation models. It is directly happening on the anode and implies the direct switch of electrons from the anode to the reducers. There are often two challenges for direct electrochemical oxidation, as it limits the rate of uptake of contaminants from the bulk solvent toward the anode and suppresses the electrochemical oxidation method due to the passivation of the cathode surface [73]. For indirect EO, in situ electro-generation of the oxidizing species takes place on the anode surface. This oxidizer is used to partially or completely decontaminate without producing by-products. The EO (electrochemical oxidation) oxidizes the S^2-^ ions into the elemental sulfur of the industrial effluent. Electrochemical sulfide oxidation encompasses a wide spectrum of direct and indirect sulfide oxidation processes that can occur cumulatively. In terms of indirect oxidation, sulfide is oxidized via anodically generated intermedial oxidants (OH∙, O_2_, Cl_2_). The oxidation products can be a combination of S^0^, polysulfides, sulfate (SO_4_^2-^), and thiosulfate (S_2_O_3_^2-^). The oxidation products are affected by the electrode material employed as well as operational parameters such as sulfide content, anode potential, convection, and pH [73,74]. It also contributes to the recovery of metals through the oxidation of metal chelates to free metal ions than by methods of reduction [75]. Electrochemical reduction occurs at the cathodes using electrons provided by external electrical energy, causing a decrease in the valence states of oxidizers to the cathode. This treatment of wastewater includes predominantly precious metal electroplating and electro-chemical denitrification [22,76,77]. Electrochemical denitrifications have been successfully used to retrieve precious metals by reducing liberated metal ions to elementary shapes over a longer period. Electrochemical denitrification makes it possible to converts nitrates and nitrites into ammonium, This can be retrieved further as ammoniacal gas via stripping or concentration using membrane methods [78,79].

### 4.2. Electrochemical coagulation

Electrocoagulation is a process that can generate metallic oxides by electrochemical in situ, destabilizing and clumping particles, or precipitating and adsorbing dissolved contaminants such as traditional chemical coagulation processes. In the early stages, electrocoagulation processes using Fe, Al, and Mg anodes were used to remove and recover P from wastewater. Metals may also be collected as hydroxides through electrocoagulation methods. When balanced to conventional procedures, the electrocoagulation method benefits from its simplicity, ease of operation, shorter retention time, reduced or no added chemical, quick settling of the electrogenerated flocs, lower sludge formation, and eco-friendliness [12,80,81].

### 4.3. Electrodialysis

Electrodialysis is a process of electrochemical separation technique in which anions and cations are transported in an electric field across an ion exchange membrane [82,49]. Ions with positive charges are pushed to the cathode, and ions with negative charges are moved to the opposite side of the anode. The use of electrodialysis is led by the creation of ion-exchange membranes, which results in greater recovery of water without the need for phase change, chemical, or reactions. These benefits aid the ecosystem by avoiding the use of chemical detergents and fossil fuels. Valuable nutrients and ions can be retrieved and separated in the form of concentrated fluxes using electrodialysis [83,84].

### 4.4. Microbial electrochemical technology

Microbial electrochemical technologies may now be developed as technologies that use the electrochemical interaction between microbes and electrodes. Depending on the nature and level of interaction, a distinction between primary and secondary microbial electrochemical technologies can be made [85–87]. A primary microbial electrochemical technology use processes that are related to microbial electrochemistry. For the most part, these interactions imply a transfer of extracellular electrons at the system level, during the examination of the bio-electrochemical device itself such as an MFC and MEC. Primary microbial electrochemical technologies are commonly referred to as a bio-electrochemical system [86,87].

A secondary microbial electrochemical technology makes greater use of indirect interactions, which are not part of microbial electrochemistry. This interaction includes, for instance, monitoring or adjusting the microbial response environment using electrochemical methods. It should be noted that this is an ionic bond among the electrochemical system and a microbial system should be in place to allow for such monitoring or an adaptation. This means that microbial and electrochemical systems should be close together and cannot be separated in space [86–88].

Sludge is the residue that accumulates in sewage treatment systems. Sludge is a solid, semisolid, or slurry byproduct of effluent treatment procedures. This residue is generally divided into two types: primary and secondary sludge. Primary sludge is produced by chemical precipitation, sedimentation, and other primary treatments, whereas secondary sludge is produced by biological treatments on activated waste biomass. Sewage sludge treatment can comprise a mixture of thickening, digesting, dewatering, and disposal techniques. Sludge digestion is a biological mechanism that decomposes organic materials into stable chemicals. Digestion decreases the overall quantity of solids, kills pathogens, and makes dewatering or drying the sludge simpler. Digested sludge is unobnoxious, resembling and behaving like rich garden soil. Anaerobic and aerobic digestion transform approximately half of the organic sludge solids into gases and liquids. Thermal hydrolysis, coupled with anaerobic digestion, can turn 60 to 70% of solids into gases and liquids. Not only is the quantity of the solid generated less than in normal digestion, but the increased biogas productivity can make certain WWTPs energy self-sufficient. The land is typically the end destination of treated sludge. Sludge that has been dewatered can be entombed subterranean in a landfill. It can also be spread on farming fields to benefit from its usefulness as a fertilizer and soil conditioner. After dewatering the wastewater generated contains a high concentration of nutrients and other substances, treatment plants have acquired respect as resource recovery services, overcoming their previous status as merely pollution control institutions. Newer technology and methodologies have continued to enhance the efficiency with which nutrients, energy, and other substances are retrieved from treatment plants, thereby creating sustainable markets and generating money for sludge treatment facilities. Amino acids, protein, short-chain fatty acids, enzymes, biopesticides, bio-plastics, bio-flocculants, and bio-surfactants are valuable products that can be generated from sludge processing. Incineration is a waste treatment method that involves the combustion of organic compounds found in waste products. The incineration of waste items produces, heat, ash, and flue gas. The inorganic parts of the waste largely constitute the ash, which can take the form of solid particles carried by the flue gas. Before they are released into the atmosphere, flue gases must be cleaned of gaseous and particulate contaminants. In some situations, the heat produced by incineration can be used to create electricity.

## 5. Resources from industrial wastewater

Wastewater includes several kinds of contaminants, but it also includes value-added products such as nutrients, salts, metals, chemicals, fuels, and energy [31,89].

### 5.1. Metals

#### 5.1.1. Heavy metals recovery

Despite the serious harm to human health and the ecological environment, valuable and rare metallic components of wastewater have significant market value. Because global silver demand exceeds mining output capacity necessitates recovering silver from secondary sources [90]. Depending on the chemical characteristics of metals and application requirements, EC techniques are classified as direct electrochemical reduction or free ion electrodialysis, electro-deionization, chelated ions by electrodialysis, less concentration wastewater by capacitive deionization, electrical switch ion exchange, parallel energy recovery by the bio-electrochemical system [15, 91]. Recovering heavy metals like copper, neodymium, uranium, and direct ER of tellurium, improved by oscillating electrodes [14, 40, 92; 93, 94]. Ammonium, zinc, cadmium, and nickel by bio- electrochemical and MFC technology [75,95].

#### 5.1.2. Other metals

Mass transport of direct electrodialysis is enhanced by a rotating cylinder electrode. With an appropriate concentration of Ag^+^, the Ag^+^ elimination efficiency of 99.28% and the Colombian efficiency of 21.61 microbial fuel cells were reached [90]. And the Gold and silver were recovered by Non-electrodeposition and electrocoagulation technologies. Merril-Crowe, ion exchange resins, and activated carbon in pulp are the most often used techniques for recovering Au and Ag from CN^−^ leachates; EC (electrocoagulation) is a potential new approach. The extraction of Au and Ag from cyanide solutions using electrocoagulation in three stages utilizing Al electrodes was found to be highly effective for solutions with higher CN^−^ contents. Furthermore, the kinetics of the process was dictated to be of zero-order, and the least energy utilization was attained when operating at a fixed frequency and with a spacing of 0.8 cm between electrodes.

Zero order: [A] = [A]_0_ − a k t

The initial Au and Ag values in the samples were 49.48 and 383 mg/L, respectively, according to the analysis. The elimination of up to 98.59% of Au and 99.43% of Ag demonstrated the process’s efficacy [15,80]. Potassium is obtained using redox transistor electrodialysis, and lithium is obtained through electrochemical sorption. Conductive polymers have the potential to be used as selective ion-exchange membrane substances. For K+ recovered from the water, a new redox transistor electro-dialyzer with 2 chambers divided by a PPy (polypyrrole) membrane electrode was developed. The polypyrrole membrane electrode was created by electrochemically depositing polypyrrole on a stainless-steel wire mesh. The polypyrrole membrane demonstrated electrodialysis selectivity for potassium ion in the presence of sodium ion, with a K+/Na+ secession factor of 2.10 adjunct on ion-exchange data. These findings reveal a unique redox transistor electrodialysis technology with strong potential for use in potassium recovery from effluent while consuming little energy [96; 97]. Ag^+^ ion has been decreased to Ag^+^ metallic lucid on the cathode surface. However, a rise in the concentration of Ag^+^ caused a decrease in more power density and Columbian efficacy because of Ag^+^ from the cathodic chamber to the anodic chamber, resulting in bio-anodic intoxication. Electrochemical coagulation has been used for the disposal of a gold and silver industrial effluent containing cyanide [11]. With optimum operating conditions, (99.43%) of the silver and (99.59%) of the gold were removed. Nevertheless, the challenges of electrochemical coagulation technology in recovering valuable metals remain unchanged. Separating every metal component from a mixture of precipitates is always difficult [80]. As a result of the growing use of lithium consumption in cell phone electronics and electric vehicles, and the scarcity of lithium, the reclamation of wastewater from battery reusing plants has become more and more important and cost-effective. 2- (allyloxy) methyl-12- crown-4 was utilized as a functional monomer in a surface-imprinting process to recycle Li ions from wastewater. The as-prepared Fe_3_O_4_@ SiO_2_@IIP exhibited great adsorption capacity and outstanding specificity for Lithium ions, as well as quick mass-transfer coefficients (k_f_ = 5.56 × 10^−4^ m/s). This ensured that it could retrieve and recycle Lithium(I) ions from industrial wastewater. When one ton of effluent is handled in this manner, 4.3 kg of white LiCl is recycled, resulting in 160.59 rupees financial interests for businesses. The electrochemical system consisting of one Li recovery electrode and an oxidizer generator electrode for similar lithium recovery and decay of organic contaminants was installed. To make quantitative comparisons, the Li^+^ retrieval performance of the Lithium manganese oxide/Boron doped diamond system was assessed using four parameters: (1) selectivity coefficient (K_Li/Na_), (2) lithium-ion purity, (3) lithium recovery capacity (q), and (4) lithium recovery rate.

(1) $$K_{Li/Na}=C_{Li}/C_{Na}$$

(2) $$Purity\left(\%\right)\;=\frac{CLi}{\left(CLi+CNa\right)}\times \;100$$

(3) $$q\;=\frac{R}{m}$$

(4) $$v=\frac{q}{t}$$

Where C_M_ denotes the concentrations of M^+^ ions in solution (mM), R is the volume of retrieved Li^+^ (mg), m denotes the mass of the utilized Lithium manganese oxide (g), and t denotes the time consumption (min) [96,98]. Lithium-ion responded with the cathode and produce LiMn_2_O_4_, and this is followed by the chemically adsorbed lithium liberated in a buffered solution, LiMnO_4_ was used as the anode for forming a recoverable solution with a high concentration of lithium. The findings demonstrated that a solution high in lithium with a contenting of (98.6 mol%) was achieved. A redox transistor electrolysis system fitted with a polypyrrole membrane electrode has been designed to selectively recover K^+^ [97]

### 5.2. Recovery of valuable nutrients

The discharge of nitrogen and phosphorus-containing wastewater to waterbodies resulted in serious algal bloom and eutrophication [99,100]. Phosphorus stone, an exhaustible resource, could wither out over the coming 100 years, threatening global human life and food security at risk [101,102]. The synthesis of ammonia using the Haber-broach technique accounts for 1% – 2% of electricity usage and around 1.6% of global carbon dioxide emissions. Recovery of these man-made components can offset (15%-20%) and approximately (25%) of global demand for nitrogen and phosphorus, severally [103, Tong et al., 2020]. EC precipitation in the form of ‘struvite’, ‘hydroxyapatite’, and ‘amorphous Ca_3_(PO_4_)_2_ ‘ are used to recover phosphorus from phosphorus-rich wastewater. Adsorption and chemical precipitation are the primary methods for recovering phosphate from wastewater. Chemical precipitation for PO_4_^3-^ recovery entails selecting a suitable chemical as a precipitator that can be applied prior, after, or during standard biological treatment of wastewater. The phosphorus collected by this procedure might be simply dewatered and perhaps utilized as fertilizer. As indicated in Eqs. 1 and 2, calcium and magnesium ions are often used as precipitators, reacting with phosphate to create hydroxyapatite = HAP (Ca_5_(OH)(PO_4_)_3_) and struvite = MAP (MgNH_4_PO_4_∙6H_2_O), respectively.

(5) $$5Ca^{2+}+\;3P{O_{4}}^{2-}+OH^{-}\to Ca_{5}\left(OH\right){\left(PO_{4}\right)}_{3}↓$$

(6) $$Mg^{2+}+P{O_{4}}^{3-}+N{H_{4}}^{+}+\;6H_{2}O\to MgNH_{4}PO_{4}\times \;6H_{2}O↓$$

Struvite, which was retrieved via various methods, could be directly put into the soil as a fertilizer, whereas hydroxyapatite could be recovered by the phosphate industries [104,105]. Electrochemical stripping and acid trapping are high-tech processes for recovering ammonia from nitrogen-rich wastewater. Electrodialysis and capacitive deionization are utilized for concentrated nutrients in low-concentrated effluent [106].

#### 5.2.1. Phosphorus

As a result of the higher pH gain at the cathode caused by water electrolysis in a separated electrochemical cell, PO_4_^3-^ precipitation from the nano-filtration concentrated was triggered. Effective pH-incumbent recovery efficiency showed that (70%-95%) PO_4_^3-^ has been retrieved at a pH ranging from 8 to 10. Also, the formation of calcium phosphate bubbles on the cathode surface has been avoided because of the cathode’s in-situ generation of hydrogen bubbles. The air-fuel cell Mg is a hopeful technique for simultaneously recovering electricity from artificial wastewater without the addition of chemicals [107]. In contrast to orthophosphate, hypophosphite is not directly collected from wastewater. The majority of the hypophosphite was retrieved via precipitation and oxidation procedures. To begin, hydroxyl radicals were used to oxidize hypophosphite to phosphate and phosphite via the electro-Fenton method. Second, the phosphate was retrieved by depositing high purity FePO_4_. The impact of current intensity, starting pH, and hydrogen peroxide concentration on hypophosphite retrieval was investigated. As a result, higher voltage intensity and hydrogen peroxide concentration enhanced hypophosphite recovery. Without the injection of hydrogen peroxide, the recovery of H_2_PO_2_^−^ was only 26.61%. The recovery of H_2_PO_2_^−^ improved to 59.6% when the hydrogen peroxide concentration was increased to 90 mM. Using X-ray diffraction, Scanning Electron Microscopy with Energy Dispersive X-Ray Analysis, High-resolution transmission electron microscopy, Fourier-transform infrared spectroscopy, and X-ray photoelectron spectroscopy technologies, it was found that the deposition was high-purity FePO_4_. Moreover, in reaction with ferric, advanced-clean FePO_4_ is generated in form of a testimony. 59.6% of the hypophosphate was retrieved in the method. Iron sludge containing Phosphorus from the primary sedimentation of the WWTP is a critical origin of phosphorus. Electro-fermentation was performed to decrease sludges and extract resources [108]. The application of 0.5 to 1.0 V to the electro-fermentation method may significantly increase the disintegration of the phosphorus from (8% to 56%) after 4 days of processing. As a result, a high phosphorus solution was retrieved as a fertilizer.

#### 5.2.2. Nitrogen

For a long time, electrochemical stripping was used to retrieve ammonium from anaerobic digestion. The electrical field between the cathode and anode changes ammonium from the anode compartment through the ion exchange membrane to the cathode compartment. Because of the elevated pH in the vicinity of the cathode and the stripping of the hydrogen, ammonium was dehydronation into volatile ammonia gas, which had been adsorbent with acid. The present density has affected ammonia flow and retrieving capacity. 57.5% of total nitrogen was retrieved in the form of ammonium sulfate, much like in real urine. In contrast to ammonium, nitrate from wastewater is difficult to volatilize with a view to recovery. Wan et al. recently proved the probability of dissimilatory NO_3_^−^ decrease to NH_3_ in an MFC using mixed electroactive bacteria. Ammonia, NO_2_^−^, and NO_3_^−^ are the three forms of active nitrogen found in wastewater. Ammonia is a fundamental nitrogen fertilizer type that can be simply isolated from water due to its volatility and/or electrical mobility. Several techniques for recovering ammonia from wastewaters have been documented, involving ion exchange, forward osmosis, and stripping. Bio electrochemical technologies were recently noted to retrieve ammonium nitrogen from wastewaters by employing limited energy, which also helped to decrease ammonia toxicity in anaerobic digestion. Ammonium was discovered to build in the cathode chamber through migration and dispersion before being collected by an acidic medium linked to microbial fuel cells. The catholyte’s high pH (more than 12) further converted ammonium into ammonia gas, resulting in 96% NH_4_^+^ recovery from artificial reject water and 87.6% in concentrated hydrolyzed urine utilizing MECs. As a fertilizer, the retrieved (NH_3_) is a possible nutrient for agricultural production. In several anoxic or limited O_2_ environments, NO_3_^−^ reduction and denitrification happen together. Several kinds of electroactive bacteria, particularly those relating to the genera Shewanella and Geobacter, have been identified as dissimilatory nitrate reduction to ammonia bacteria. The electroactive biofilm can be used as a dissimilatory nitrate reduction to ammonia system to change all NO_3_^−^ into ammonium prior retrieval, enabling NH_3_^–^ N recycling a possibility [109]. A stable and recoverable ammonia process was identified, with carbon/nitrogen ratios ranging from 0.5–8.0. contrary to traditional denitrification in microbial electrochemical, the efficiency of dissimilar Nitrate/nitrite reducing to ammonium could reach a peak of (44%). The bio-electrochemical ammonium technique demonstrated the availability of converting the oxidation state of nitrogen such as NO_3_^−^ and NO_2_^−^ into NH_4_^+^ for subsequent recovery. Most significantly, in contrast to the electrochemical reduction of nitrates, GHGs (greenhouse gasses) emissions, like nitrogen oxide generation, can be removed during the dissimilar Nitrate/nitrite reduction to ammonium process [J. 110, 111].

1. Others

A hybrid cation-exchange membrane electrolysis/magnesium to recover K_3_PO_4_, a crystallization procedure was produced NH_4_^+^ – N and CL^−^ from nanofiltration concentrate concurrently and also useful potassium ions. The projected combination process could eliminate (99%) of NH_4_^+^ – N and at the same time recover potassium.

(7) $$Cl^{-}\to Cl^{.}+\;e^{-}$$

(8) $$Cl^{.}+Cl^{.}\to Cl_{2}\left(g\right)\;↑$$

(9) $$Cl_{2}\left(aq\right)\;+\;H_{2}O\to HClO+HCl$$

(10) $$Organic+HClO\;\;\to intermediates\;\to \;CO_{2}+\;H_{2}O$$

(11) $$2NH_{3}-N+\;3HClO\;\to \;N_{2}+\;3H_{2}O+\;3H^{+}+\;3Cl^{-}$$

The initial stage of the combination process investigated in this work, cation-exchange membrane electrolysis (CEME), was utilized to concurrently eliminate organic contaminants from nanofiltration concentrates and retrieve Cl^−^ ions through electro-generated gaseous Cl. Furthermore, the created gaseous chlorine doesn’t have to be discarded, but it can be utilized onsite as a handy agent to discolorize colored effluent. The second part of this research involves the extraction of potassium from remediated nanofiltration concentrations using electromigration and subsequently potassium retrieval by a MgKPO_4_ ∙ 6H_2_O crystallization technique. Slow-release fertilizers containing MgKPO_4_ ∙ 6H_2_O are important and limited

Mg^2+^ + PO_4_^3-^ + K^+^ + 6H_2_O MgKPO_4_ ∙ 6H_2_O↑

Approximately 53% of the k (from 2762 mg/L to 1389 mg/L) was eliminated through precipitation of (MgKPO_4_ ∙ 6H_2_O), a good buffered fertilizer. The results showed that potassium can be recovered from nanofiltration concentrates in the form of MgKPO_4_ ∙ 6H_2_O precipitate in the cation-exchange membrane electrolysis system [112]. To conserve energy, the microbial fuel cell was employed to retrieve nutrients from urine-containing wastewater [113,114]. As with late studies, hydrolysis of urea occurred through a bio-electrochemical method, and ions migrating due to a self-created electrical field. The findings indicate that (42%) of the total nitrogen (37%) of phosphate was collected in the central chamber. Besides, the findings suggest that (97%) of COD (chemical oxygen demand) was eliminated, resulting from the recovered solution with undetectable micropollutants.

### 5.3. Recovery of Sulfur and Hydrogen

SO_4_^2-^ and S^2-^ have both created a number of environmental issues, including corrosiveness, poisoning to the marine world, and offensive smell. Sulfide can be readily oxidized and turned into a sulfur ion, which is a great cathodic material in the lithium-sulfur battery [115]. A new integrated strategy of biological (sulfate-reducing bacteria) and electrical oxidation method has been developed for the recovering of Sulfur by minimizing the content of sulfate polluted pond water. *Bacillus licheniformis, Stenotrophomonas maltophilia*, and *Bacillus cereus*, as well as a smaller proportion of naturally found anaerobes, have also been involved in this procedure through using peptone and glucose as sources of energy in the wastewater for the reduction of SO_4_^2-^ to S^2-^ and formation of transition metal sulfide residue. Sulfide residues were organically recovered from sulfate-polluted water near the Na_2_S_2_O_6_ production business at basic pH (9.25). The electrochemical procedure turned the biological metal sulfide residues into the alkaline metal sulfide, which was then oxidized to Sulfur. At a lower current density of 20 mA/cm^2^, a Ti-TiO_2_/IrO_2_/RuO_2_ combined metal oxides coated standard electrodes was used as an anode in an electrochemical sulfide oxidation method. Using a typically mixed metal oxide anode and an electrochemical technique, 70% of the Sulfur was recovered. X-ray diffraction was used to confirm the recovery of Sulfur. Energy-dispersive X-ray analysis revealed that the Sulfur was pure (100%). The pH of the solution is critical in the sulfide oxidation reaction. At the cathode chamber, NaOH has also been recreated. Sulfur retrieved was tested as a cathode in an energy storage system (Li-S battery). The CV (cyclic voltammetry) and charge-discharge profiles showed that the retrieved Sulfur has been used as an intense cathode substance in a Li-S battery [27,73]. Because of its less carbon, high energy, and renewable characteristics, H_2_ was identified as pure energy for sustainable development worldwide [116,117]. The Microbial electrolysis cell is a microbial electrochemical technique that enables anaerobic bacteria consortiums to transform biodegradable waste into electricity. The electrons are then shifted to the cathode, where they are reduced to protons for hydrogen generation with the use of a lower external voltage (0.2–0.8 V) to exceed the thermodynamic barriers of water electrolysis. The maximum rate of hydrogen generation was 168.01 ± 7.01 mL/L/d, with a hydrogen yield of 5.14 ± 0.22 mmol/kg COD (3000 mg COD/L, 1.0 V), while the maximum cathodic hydrogen recovery and energy efficiencies were 74.24 ± 0.11% and 120.56 ± 17.45%, consequently. In duplicate reactors with minor changes, hydrogen gas was created. Because of the electrolysis, hydrogen generation became unsteady at 1.2 V. The higher extraction efficiencies are ascribed to a combination of effective microbial electrochemical biodegradation and activated carbon adsorption, and the in situ produced hydrogen can be utilized for biocrude oil improvement on-site [118,119]. The voltage applied to microbial electrolyte cells is 2 times smaller than that used in electrochemical water splitting. Minimizing cathode activation, concentration resistors, and ohmic is essential to achieve effective H_2_ yield [120–121, Y. 122]. A cathode synthesized through the in-situ growth of acid-rich Co_3_(PO_4_)_2_ nanoarrays on the Ni foam matrix showed exceptional electrolytic conductivity [123]. Because of the greater active electrochemical surface and the lower resistance to charge transfer of phosphating cobalt-nickel foam, the production rate of hydrogen improved three times as compared to bare nickel foam and platinum/carbon obtained. The higher total energy recovery of the phosphating cobalt-nickel foam-based microbial electrolysis cells reached (40 ± 4.0%), which was also 3 times greater than that of the cathode with platinum/carbon.

### 5.4. Recovery of organics and chemicals

The entire mineralization of organic matter into carbon dioxide leads to waste and a greenhouse effect. Numerous investigation groups have tried to retrieve valuable goods through EC methods, admitting methane, VFAs, and others [124]. Chemically enhanced primary sedimentation is incorporated into the process. Organic contaminants are removed more efficiently at a wastewater treatment plant, but organic-rich sludge is left behind. These sludges provide an excellent source of precious components. Electro-fermentation has shown the viability of treating organically rich sludge and recovering precious resources [125]. A 2 chamber electro-fermentation cell separated by a cation exchange membrane has been established to retrieve VFAs from the sludge [108]. Electrostimulation contributed to the richness of functioning microbe populations, which leads to greater purity of the volatile fatty acids recovered from the sludge supernatant compared to the single-chamber fermenter. Methane was recovered at a lower temperature in the electro-aided anaerobic membrane bioreactor method, which utilized CNTs and hollow fiber membrane as a cathode [126]. Electro-aided-membrane CNTs with hollow fibers had both membrane filtration and cathode functions. Because of the large amount of methanomicrobic and methanogen using hydrogen, an anaerobic electro-aided membrane bioreactor may generate more than 111.12 ml g^−1^ VSS d^−1^ of methane. Recovering alkali from high-grade alkaline solution has been judged in electrodialysis batteries. A (9%) solution of sodium hydroxide was filled into the centralized chamber, whereas the diluted solution of (3%) sodium hydroxide was employed in the electrode cell. The electrodialysis technique has demonstrated current efficiencies of approximately (60%) [127]. The electrochemically switched ion exchange system may also be capable of producing a sodium hydroxide solution at pH 12.8 using an applied less voltage. Power ingestion of 2,083 x 10^−3^ kWh mol^−1^ was needed to retrieve the sodium ions during the process. The Na^+^ ion-exchange capacity ($q_{t}$) in mg g^1^ is determined as follows:

$$q_{t}=C_{0}-C_{t}\times V$$

Where, C_0_ is the initial concentration of sodium ions (mg L^−1^), C_t_ is the concentration of sodium ions at time t, V is the amount of treated solution (L), and m is the average weight of ferric ferricyanide nanoparticles placed on the electrode (g).

The concentration of sodium ions was quite high during the first 30 minutes and then subsequently declined to an equilibrium value at around 120 minutes, indicating that ferric ferricyanide exhibited a great attraction for sodium ions. The mass of segregated phenol changed as sodium ions were inserted into the ferric ferricyanide-coated electrode. More than 27.7% of phenol was segregated at various concentrations, denoting that the Electrochemically switched ion exchange technique, as an electrochemical process, could eliminate phenol in C_6_H_5_NaO solution. Depending on the conservation of electric charge, the cathode was pushed by a voltage to form OH ions, allowing sodium hydroxide to be extracted from the reestablished solution. The concentration of sodium ions and the pH of the suspension change throughout electrode regeneration. Over 98.0% sodium ions were discharged into the suspension demonstrating that the electrode had been reestablished sufficiently to allow this electrode to be empty for the next sodium ion insertion. Depending on the conservation of electrical charge, the cathode was driven by a voltage to produce OH^−^ ions, allowing sodium hydroxide to be extracted from the replenished solution [128].

### 5.5. Heat and others

As a result of interfacial joule heating, the electrolyte temperature increased during the electrolysis process [129]. The effective recovery of heat by using the effluent remediation technique was tested for determination through the manufacture and design of an electrochemical reactor. The use of ohmic heat in a mixed technique to eliminate salt without devouring outside power has been investigated recently. The Electrochemical oxidation – direct contact membrane distillation (DCMD) hybrid method for pollutant anodic treatment followed by Ohmic heating-driven distillation electrochemical oxidation was carried out with a boron-doped diamond (BDD) anode with a vast potential range (−1.25 to +2.3 V_SHE_) in the existence of SO_4_^2-^ as an electrolyte, quickly treating the organics with various oxidation forms: water- or anion-derived oxidants are used for direct electron abstraction and oxidation. The Electrochemical oxidation – direct contact membrane distillation hybrid method was related to the direct contact membrane distillation method in terms of (1) efficiency in removing 8 organics, which include benzoic acid, acetaminophen, cimetidine, caffeine, nitrobenzene, linuron, triclosan, and sulfamethoxazole, and (2) membrane wetting resistance when SDS (sodium dodecyl sulfate) is present. To evaluate the defouling activity of anodically produced SO_4_^• –^ and persulfate. In the EO – DCMD hybrid procedure, the limit of water flux recovery was assessed. while running it in the presence of alginate as a model material to generate membrane fouling; alginate, a natural polysaccharide composed of inconstancy sequences of guluronate and mannuronate, has frequently been employed to imitate organic fouling in membrane technology. Finally, the Electrochemical oxidation – direct contact membrane distillation hybrid process was evaluated in actual flue gas desulfurization effluent without an outer source of heat energy or electrolytes for sequential anodic organic oxidation followed by desalination based on distillation [130]. In the electrochemical oxidation field of the hybrid processes involving electrochemical oxidation and DCMD, the temperature of the SO_4_^2-^ electrolyte rose to 70°C. The warmed solution was then fed in the DCMD for pure water. During this time, an in-situ production of sulfate radicals has been caused by ohmic heating, which eliminated the clogging of membranes by decomposition of enriched organic materials. The most obvious benefit of electrochemical oxidation DCMD was the removal of outside thermal power and electrolyte regeneration. The electrolyte needed in electrochemical oxidation was revitalized by DCMD, whereas the thermal source of the DCMD could be provided by electrochemical oxidation. To retrieve phosphorus from Fe^3+^ sludge produced during chemically enhanced primary sedimentation, sulfide must be added, but FeS precipitation must be left. Recovery of iron and sulfur from this ferrous sulfide sludge through electrochemical techniques has considerable economic advantages.

2FePO_4(s)_ + 3H_2_S → 2FeS_(s)_ + S^0^_(s)_ + 2H_2_PO^−^_4_ + 2 H^+^

Sulfide additions may effectively recover P from FePO_4_ sludge, achieving 70 ± 6% recovery at a sulfur/iron stoichiometric molar ratio of 1.5 and rising to 92% recovery at a sulfur/iron molar ratio of 2.5. This was verified when the sulfur/iron molar ratio obtained in the solid-state was measured to be around 1. 5 moles. It was also discovered that the liqule economic advantid and solid phases separated quicker. It was later shown, however, that this was because of some hydrogen sulfide loss throughout the acidic digesting step [131]. Pivotal responses included electrochemical oxidation from ferrous sulfide to S ion and soluble ferrous ions, oxidation of ferrous ions with ferric oxyhydroxide, and subsequently, acid dissolution of ferrous oxyhydroxide into free ferric ions.

Anode Oxidation Reaction

Redox Potential

(1) FeS →Fe^2+^ + S^0^ + 2e^−^ + 0.06^I^

(2) FeS_(s)_ + 4H_2_O →Fe^2+^ + SO_4_^2-^ + 8 H^+^ + 8e^−^ −0.09^II^

(3) Fe^2+^ + 3H_2_O→Fe (OH)_3_ + 3 H^+^ + e^−^ (at pH>3) 0.51^III^ (at pH 3.0)

(4) Fe^2+^→Fe^3+^ + e^−^ (at pH<3) +0.771^IV^

Cathode Reduction Reaction:

(5) S^0^ + 2e^−^→S^2- −0^.476^I^

The method relies on the EO of sulfide to S^0^ and partly sulfate, resulting in the release of soluble Fe^2+^ in the solution. As the pH lowers to 3 owing to the acidity caused by the Fe (OH)_3_ and SO_4_^2-^ production, soluble Fe^2+^ is oxidized to Fe (OH)_3_, and then to free ferric ions. Because the produced S^0^ is attached to the surface of the anode, it may be reduced back to S^2-^ upon polarity change of the electrode, with electricity serving as its only input value. Carbon-based electrode materials were chosen for this work because of their established reactivity with FeS, cheap cost, and widespread accessibility. Higher iron recoveries were obtained when actual FeS suspension was fed into the procedure (60%) compared to synthetic FeS solution (41%) on graphite granules [132]. Thus, (60 ± 18%) soluble iron and (46 ± 11%) sulfides were anode and cathode and anode chambers regenerated by electrochemistry, severally. When handling the actual ferrous sulfide suspensions resulted in the peak flow compaction of 4.5 ± 9.5 m^−2^ and minimal power uptake of 0.5 ± 2.4 kWh kg Fe^−1^ respectively was obtained. Table 2 shows the recovery of valuable metals, nutrients, and chemicals from wastewater.

**Table 2.** Recovery of valuable metals, nutrients, and chemicals from wastewater

Sr.No.	Recovered resource	Technique used	References
1.	Silver	Electrically switched ion exchange technique, Non-electrode deposition method.	80**,al. (2020)**
2.	Gold	Electrocoagulation	Carrillo et al., [15]
3.	Lithium	Electrochemical sorption	91
4.	Potassium	Redox transistor electrodialysis	97
5.	Phosphorus	Electrochemically generated precipitation: Calcium phosphate	18
6.	Phosphate	Electro-hydro modulation, electrochemically generated precipitation of calcium phosphate	Perera et al., [104)
7.	Nitrogen	Electrochemical stripping, acid absorption, integrated membrane electrode	133
8.	Nitrogen	Capacitive deionization	106
9.	Total nitrogen, Phosphate	Microbial fuel cell	114
10.	Sulfide, Elemental sulfur	Electrochemical oxidation	134
11.	Volatile fatty acids	Electro-fermentation, cation exchange membrane	108
12.	Methane	Anaerobic electro-assisted membrane bioreactor system, nanotubes hollow fiber membranes	126
13.	Na^+^ ion	Electrodialysis, electrochemically switched ion exchange	128
14.	Fe & S from FeS	Electrochemical	Mejia et al., [2014)
]15.	Heat	Electrolysis process, electrochemical reactor	130

## 6. Bottlenecks and Perspectives

Despite substantial progress in resource retrieval from effluent using EC techniques, moving from the status of ‘promising technique’ to ‘practical technique’ remains a challenge. Single electrochemical reduction is a decent way to transform dissolved metal ions into metal deposits, but it’s not enough to break chemical bonds in metal complexes. By improving the operational characteristics, electrode materials, electrolysis process, and mass transport, the electrochemical reduction’s efficiency can be increased. Electrochemically switched ion exchange, a membrane-based EC technique, has been effectively used to retrieve many heavy metal ions with great selectivity while requiring a lot of energy. Phosphate can be transformed into value-added fertilizers using electrochemical precipitation. However, lowering the cost of chemical addition while improving fertilizer purity remains a major issue that requires immediate attention.

Furthermore, cathode fouling caused by precipitate deposition decreases performance and raises energy consumption. Electrochemical stripping is a useful method for converting free NH_4_^+^ ions to gaseous NH_3_, which can then be utilized or transformed to (NH_4_)_2_SO_4_. The addition of transition metal carbides to an electrochemical stripping system will improve performance while lowering energy consumption. Hydrogen is emitted synchronously at the cathode during the electrochemical wastewater decontamination phase because of water splitting. Although the production of hydrogen in conjunction with the reduction of contaminants is appealing and considered promising, the high energy demand is still a barrier to its widespread adoption. EC should advance in the areas of energy conservation and several-resource recovery. When dealing with effluent containing diverse constituents and varying end-user criteria, a single technique is often inadequate to obtain desirable goods.

The electrodes are the site of metal recovery in an EC recovery system. It is critical to design electrode material or electrodes with higher density active sites and excellent selectivity, which won’t only save money but also allow metals to be recovered individually from effluent. This includes a long life-cycle, increased depth of discharge, traditional applications, increased energy and power density, broad operating temperature ranges, and lower costs while boosting system safety and dependability.

Selective pre-separation of important components from non-value components is necessary to increase product recovery capacity and clarity. The use of an electrochemically active membrane device for several-resources retrieving from effluents, such as electric energy, water, and valuable goods, may be promising. Stable electricity supply has been a bottleneck in remote areas, limiting the use and growth of EC methods. Exploring solar/wind energy or combining fuel cell technology to create self-powered devices appears to be a viable option. Waste material can be processed into a range of value-added goods using MESs, which are among the appropriate platforms for recovering energy and resources. The use of EC techniques to recover resources from small-size or decentralized effluent treatment plant appears to be a good fit.

## 7. Conclusions

Electrochemical techniques like electrochemical oxidation, electrochemical reduction, electrodialysis, electrocoagulation, and microbial electrochemical techniques were extensively studied to recover valuable products such as nutrients, salts, metals, chemicals, compounds, and energy in terms of precipitation, deposition, and concentrated mixture. Using MESs, which are one of the most appropriate platforms for recovering energy and resources, effluents may be transformed into various value-added products. Electrochemical techniques can be advanced to conserve energy and recover multiple resources from wastewater. When it comes to waste containing various components, single technique is often insufficient to achieve suitable products. Subsequent research is needed to concentrate on reducing the system’s cost, improvement of product grade, and developing a smart system. Furthermore, self-sustaining, cost-effective, scalable, and efficient electrochemical systems for remote areas and decentralized wastewater are required to be developed. Furthermore, traditional and new wastewater treatment techniques were thoroughly studied, with a review of the benefits and drawbacks of each technique. Overall, the prospects for wastewater-based resource recovery through electochemical techniques are encouraging, as long as the process feasibility and long-term sustainability are assured.

## Highlights

- Prospects for effluents based resource recovery employing electrochemical techniques are promising.
- Microbial electrochemical technologies are appropriate platforms for recovering resources from effluents.
- Benefits and drawbacks of wastewater treatment techniques have been discussed.
